# The comparison of contrast-enhanced ultrasound and gadoxetate disodium-enhanced MRI LI-RADS for nodules ≤2 cm in patients at high risk for HCC: a prospective study

**DOI:** 10.3389/fonc.2024.1345981

**Published:** 2024-05-07

**Authors:** Zhengyi Qin, Yan Zhou, Xiang Zhang, Jianmin Ding, Hongyu Zhou, Yandong Wang, Lin Zhao, Chen Chen, Xiang Jing

**Affiliations:** ^1^ Department of Ultrasound, Tianjin Third Central Hospital, Tianjin, China; ^2^ Tianjin Key Laboratory of Extracorporeal Life Support for Critical Diseases, Tianjin, China; ^3^ Artificial Cell Engineering Technology Research Center, Tianjin, China; ^4^ Tianjin Institute of Hepatobiliary Disease, Tianjin Third Central Hospital, Tianjin, China; ^5^ School of Medicine, Nankai University, Tianjin, China; ^6^ Department of Radiology, Tianjin Nankai Hospital, Tianjin, China; ^7^ Department of Radiology, Tianjin Third Central Hospital, Tianjin, China

**Keywords:** Liver Imaging Reporting and Data System, contrast-enhanced ultrasound, contrast-enhanced magnetic resonance imaging, hepatocellular carcinoma, EOB-MRI

## Abstract

**Objectives:**

To investigate the consistency of LI-RADS of CEUS and EOB-MRI in the categorization of liver nodules ≤2cm in patients at high risk for HCC.

**Methods:**

Patients at high risk for HCC with nodules ≤2cm who underwent CEUS and EOB-MRI in our hospital were prospectively enrolled. The CEUS images and EOB-MRI imaging of each liver nodule were observed to evaluate inter-observer consistency and category according to CEUS LI-RADS V2017 and CT/MRI LI-RADS V2017 criteria double blinded. Pathology and/or follow-up were used as reference standard.

**Results:**

A total of 127 nodules in 119 patients met the inclusion criteria. The inter-observer agreement was good on CEUS and EOB-MRI LI-RADS (kappa = 0.76, 0.76 p < 0.001). The inter-modality agreement was fair (kappa=0.21, p < 0.001). There was no statistical difference in PPV and specificity between CEUS and EOB-MRI LR-5 for HCC, while the difference in AUC was statistically significant. We used new criteria (CEUS LR-5 and EOB-MRI LR-4/5 or CEUS LR-4/5 and EOB-MRI LR-5) to diagnose HCC. The sensitivity, specificity, and AUC of this criteria was 63.4%, 95.6%, and 0.80.

**Conclusions:**

CEUS and EOB-MRI showed fair inter-modality agreement in LI-RADS categorization of nodules ≤2 cm. The inter-observer agreement of CEUS and EOB-MRI LI-RADS were substantial. CEUS and EOB-MRI LR-5 have equally good positive predictive value and specificity for HCC ≤ 2cm, and combining these two modalities may better diagnose HCC ≤ 2 cm.

**Clinical Trial Registration:**

https://clinicaltrials.gov/, identifier NCT04212286.

## Highlights

- CEUS and EOB-MRI showed fair inter-modality agreement and substantial inter-observer agreement in LI-RADS categorization.
- CEUS and EOB-MRI LR-5 have equally good positive predictive value and specificity for HCC ≤ 2 cm, and combining these two modalities can better diagnose HCC ≤ 2 cm.

## 1 Introduction

Hepatocellular carcinoma (HCC) is the most common primary malignant tumor of the liver (1). Although the mortality rate is high, early detection and treatment can obtain a good prognosis (2). In high-risk patients, lesions with the typical enhancement pattern of HCC on imaging can be diagnosed noninvasively without further requirement for histopathological confirmation (2), which shows the importance of contrast-enhanced imaging. Compared with contrast-enhanced computed tomography/magnetic resonance imaging (CECT/MRI), contrast-enhanced ultrasound (CEUS) has the advantage of lower cost, better temporal and spatial resolution, and comparable or even better diagnostic performance than CECT/MRI (3, 4). On the other hand, the hepatobiliary phase of ethoxybenzyl-enhanced magnetic resonance imaging (EOB-MRI) could increase the diagnostic performance of HCC, especially early HCC (5–7).

The Liver Imaging Reporting and Data System (LI-RADS), first published by the American College of Radiology (ACR) in 2011, CT/MRI LI-RADS, was updated based on new evidence-based knowledge and feedback. The CEUS LI-RADS came out in 2016 and was updated in 2017. Because of the difference in contrast agents and techniques between CEUS and CT/MRI LI-RADS, the major features and ancillary features are different. Therefore, a comparison of CEUS and EOB-MRI LI-RADS, two highly effective imaging diagnostic tools, is rarely reported. Recently, an intra-individual comparative study on EOB-MRI and CEUS LI-RADS (8) reports that the inter-modality agreement of those LI-RADS was moderate (kappa=0.449). However, their study was retrospective and focused more on larger nodules (mean diameter of 4.8 ± 3.6cm). For smaller nodules, the imaging features may be different. The purpose of this study was to investigate the consistency of LI-RADS of CEUS and EOB-MRI in the categorization of liver nodules ≤2 cm in patients at high risk for HCC.

## 2 Materials and methods

### 2.1 Patients

This study was prospectively registered at ClinicalTrials.gov (NCT04212286). This study was approved by the ethics committee of our hospital. Patients at high risk of HCC who received both CEUS and EOB-MRI in our center from November 2019 to December 2022 were enrolled in this study. A total of 119 patients with 127 nodules were included. The diagnosis of malignant lesions was based on pathology (including surgical resection and ultrasound-guided biopsy). The diagnosis of benign lesions was based on ultrasonic-guided biopsy and/or follow-up (nodule diameter increase <50% in 6 months and no change in enhancement pattern) as the reference standard. Inclusion criteria were as follows (1): patients at high risk factors for HCC, such as cirrhosis, chronic hepatitis B, and a history of HCC; (2) age of 18–80 years old; (3) routine CT/MRI/US scan found nodules ≤2 cm; (4) the number of lesions in each patient ≤3; (5) both CEUS and EOB-MRI were performed within 1 month; and (6) patients’ informed consent was obtained. Exclusion criteria were as follows: (1) nodules without a definite diagnosis; (2) the nodules had received treatment, including local ablation therapy or TACE; (3) severe heart, lung, liver, and renal insufficiency; (4) lactating and pregnant women; and (5) those who were assessed by the researchers as not suitable for inclusion.

### 2.2 CEUS techniques

US images were obtained by Philips EPIQ 7 ultrasound system (Philips Medical System, Bothell, WA, USA) equipped with a C5-1 (1.0–5.0 MHz) convex array probe, pulse inversion imaging (PI) software, and mechanical index 0.04–0.08, or by Acuson S3000 ultrasonic diagnostic system (Siemens Medical Solutions, Mountain View, USA) equipped with a 6C1HD (1.0–6.0 MHz) convex array probe, contrast pulse sequencing/contrast high-resolution imaging (CPS/CHI) software, and mechanical index of 0.08–0.10. The sulfur hexafluoride microbubble (SF6) contrast agent (SonoVue, Bracco) was sufficiently mixed with 5 ml saline before bolus injection in the antecubital vein. The conventional and color Doppler US was performed to record the number, location, size, shape, pattern of internal echo, and blood flow distribution of liver nodules.

Images were captured in a standard manner, including all liver segments, with the participants placed in supine and left lateral decubitus positions. Livers were evaluated during quiet respiration. The section of nodules at the largest cross-sectional view was selected for contrast imaging acquisition. After intravenous injection of 1.2–2.0 ml of contrast agent through the antecubital vein, followed by a flush of 5 ml 0.9% sodium chloride solution, the imaging of target lesion was recorded for 60 s. After 60 s, the lesion was intermittently scanned every 1 min and recorded for 5 min to characterize washout features. All images were saved and then analyzed frame by frame.

### 2.3 EOB-MRI techniques

EOB-MR imaging was performed with Siemens Magnetom Verio 3.0-T magnetic resonance unit (Siemens Medical Solutions), using phased array surface coils. Liver MR imaging protocol consisted of in-phase and opposed-phase T1-weighted imaging, FSE T2-weighted imaging with fat suppression, and diffusion-weighted imaging. For EOB-DTPA-enhanced imaging, 0.025 mmol/kg gadoxetic acid (Primovist; Bayer Healthcare) was intravenously injected at a rate of 1.0 ml/s by using a power injector, followed by 25-ml saline flush. Arterial, portal venous, and transitional phase images were acquired at the delay time of 15–18 s, 50–60 s, and 180 s after contrast injection using volumetric interpolated breath-hold examination (VIBE) sequence. Hepatobiliary phase imaging was completed 20 min after the contrast injection.

### 2.4 Image analysis

All the radiologists were blind to the patient information, pathology results, and other imaging or laboratory examination. Two radiologists with more than 10 years of experience in liver CEUS analyzed all the CEUS images independently and evaluated the inter-observer agreement. All EOB-MRI images were reviewed by two radiologists with more than 15 years of experience in abdominal imaging. All lesions were categorized based on the CEUS LI-RADS version 2017 or CT/MRI LI-RADS version 2017. To resolve discrepancies between the two observers, images were re-evaluated together and a consensus was reached.

### 2.5 Statistical analysis

In this study, SPSS19.0 software was used for statistical analysis of the data. Quantitative data are expressed as mean ± standard deviation, and categorical variables were expressed by frequency. To evaluate the diagnostic performance of CEUS and EOB-MRI LI-RADS for HCC, accuracy, sensitivity, specificity, positive predictive value (PPV), and negative predictive value (NPV) were calculated. The diagnostic performance of two modalities was analyzed by receiver operator characteristic curve (ROC), and the area under ROC curve (AUC) was compared by Delong test. Cohen’s kappa coefficient was used to compare the inter-modality and inter-observer consistency evaluation. p < 0.05 was considered statistically significant.

## 3 Result

### 3.1 Participant characteristics

There were 127 nodules in 119 patients, including 88 men and 31 women, with an average age of 59.6 ± 8.6 (34–77) years. The 127 nodules included 82 HCC, 2 intrahepatic cholangiocarcinoma (ICC), 1 neuroendocrine carcinoma, 9 dysplastic nodules, 3 regenerative nodules, 1 hemangioma, 1 focal nodular hyperplasia, and 28 other benign nodules. A total of 96 nodules were pathologically confirmed by biopsy, and 31 were confirmed by follow-up. During follow-up in 6 months, three lesions (two of them were CEUS LR-3 and one was CEUS LR-4) turned into CEUS LR-5 and one CEUS LR-3 lesion turned into CEUS LR-4, which were all HCC confirmed by biopsy. The clinical characteristics of patients and nodules are shown in **Table 1**.

**Table 1:** Characteristics of the patients.

Characteristic	
Number of cases	119
Age (year)	59.6 ± 8.6 (34–77)
Gender	
Male	88
Female	31
Cirrhosis	113
Etiology of chronic liver disease	
Hepatic B virus infection	95
Hepatic C virus infection	10
Alcoholic liver disease	7
Other causes	7
Number of nodules	127
Single	111
Multiple	8
Size(cm)	1.4 ± 0.4(1.0–2.0)

### 3.2 Inter-observer agreement of CEUS and EOB-MRI LI-RADS

The inter-observer agreement of CEUS LI-RADS categorization was substantial (kappa = 0.76, p < 0.001, **Supplementary Table S1**). The inter-observer agreement of EOB-MRI LI-RADS categorization was substantial (kappa = 0.76, p < 0.001, **Supplementary Table S2**).

### 3.3 Inter-modality agreement of CEUS and EOB-MRI LI-RADS

**Figure 1:**
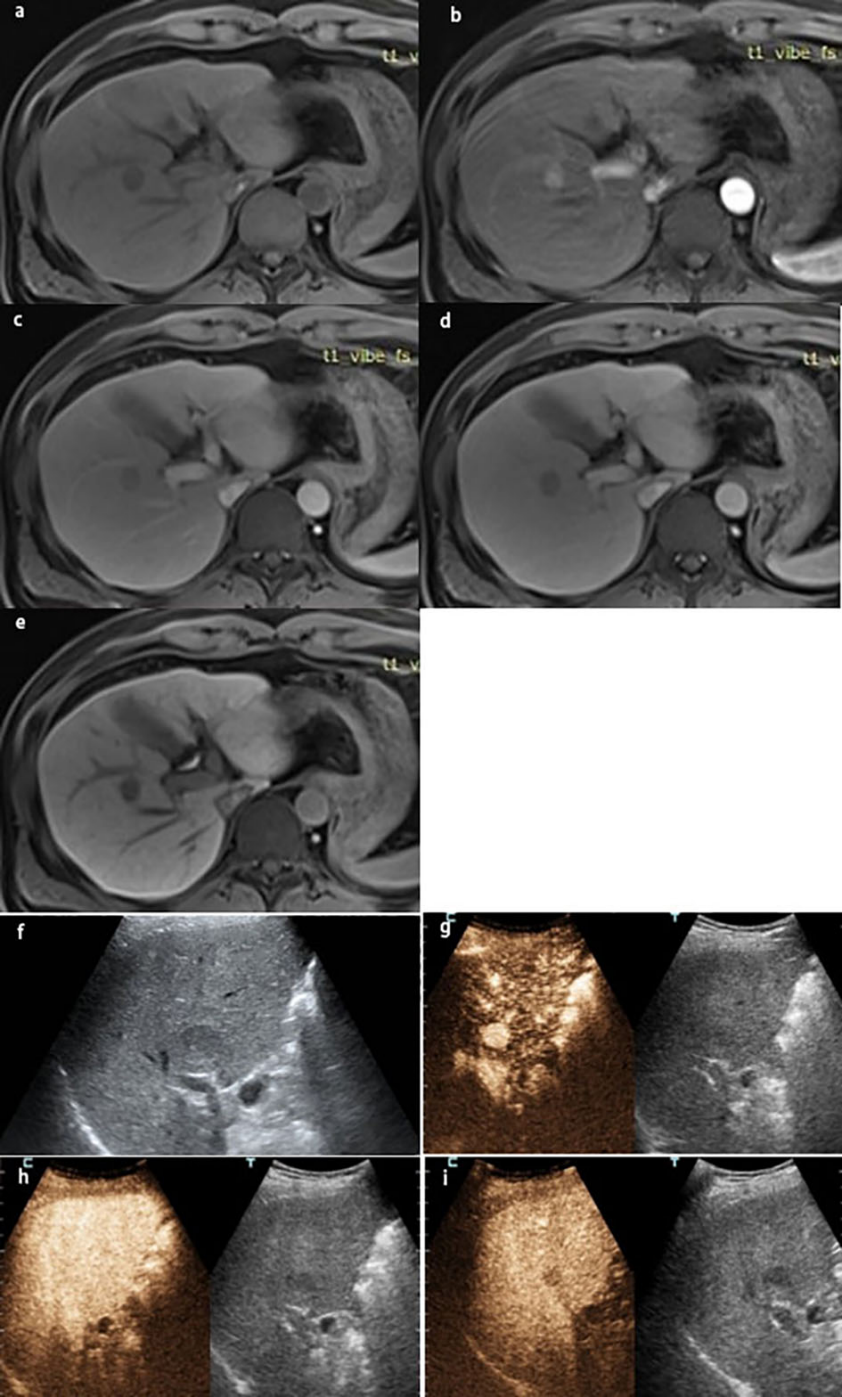
A lesion in S7-8 with a size of 1.7×1.6 cm in a 43-year-old man with chronic HBV infection and cirrhosis. The lesion was classified as LR-5 by EOB-MRI: **(A)** the lesion was hyperintensity on T1WI, showing **(B)** arterial phase hyperenhancement, **(C)** washout on portal venous phase, **(D)** hypointensity on the transitional phase, **(E)** hypointensity on the hepatobiliary phase, and **(F)** gray-scale ultrasound found a lesion on S7-8. The lesion was classified as LR-5 by CEUS. **(G)** Arterial phase hyperenhancement; **(H)** no washout on 60s; **(I)** the lesion was hypovascular on the delay phase. The pathological result by ultrasound-guided puncture was highly differentiated HCC.

**Figure 2:**
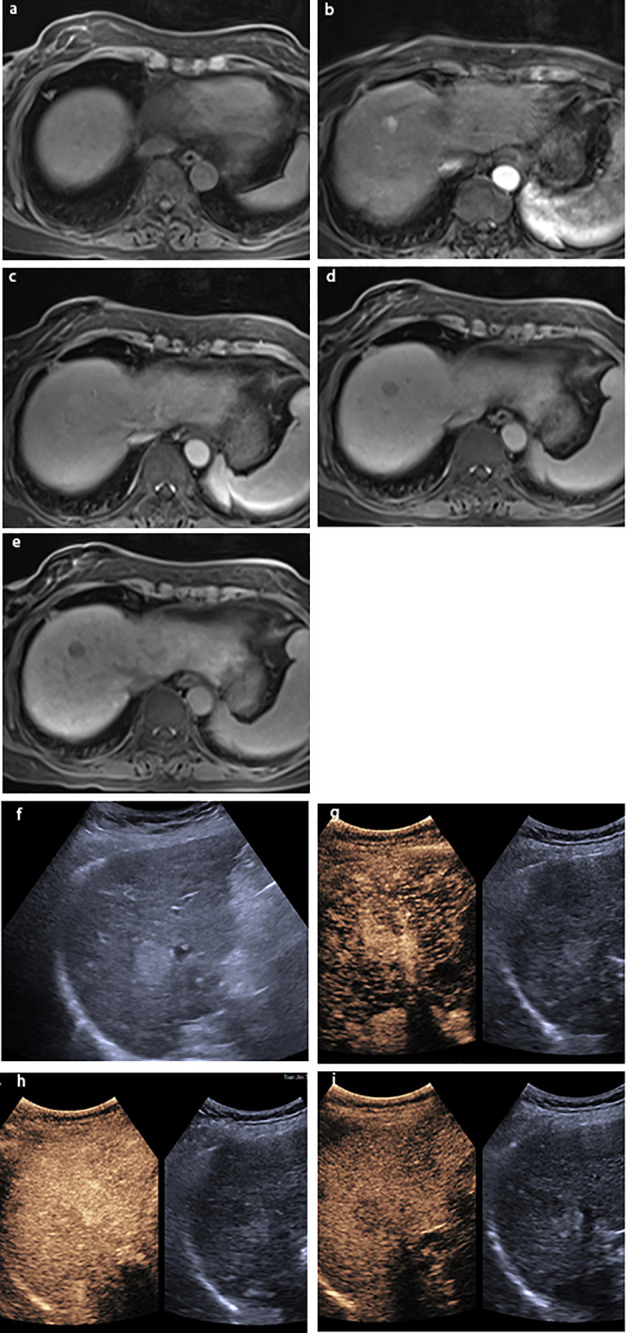
A lesion in S6 with a size of 2.0×1.6 cm in a 51-year-old man with chronic HBV infection and cirrhosis. The lesion was classified as LR-4 by EOB-MRI: **(A)** the lesion was hyperintensity on T1WI; showing **(B)** arterial phase slightly hyperenhancement, **(C)** hyperintensity on the portal venous phase, **(D)** isointensity on the transitional phase, and **(E)** hypointensity on the hepatobiliary phase; **(F)** gray-scale ultrasound found a lesion on S6. The lesion was classified as LRM by CEUS: showing **(G)** arterial phase hyperenhancement and **(H)** washout on 58 s. **(I)** The lesion was hypovascular on the delay phase. The pathological result by ultrasound-guided puncture was highly differentiated HCC.

The proportions of LR-3, LR-4, LR-5, and LR-M were 23.6% (30/127), 19.7% (25/127), 40.2% (51/127), 16.5% (21/127), and 18.1% (23/127), 51.2% (65/127), 27.6% (35/127), 3.1% (4/127) (**Table 2**), respectively, in CEUS and EOB-MRI LI-RADS. The inter-modality agreement of CEUS and EOB-MRI LI-RADS was fair (kappa=0.21, p < 0.001) (**Figures 1**, **2**). A comparison of the category results between CEUS and EOB-MRI and the pathological results and category between CEUS and EOB-MRI is shown in **Table 3**.

**Table 2:** Comparison of the category results between CEUS and EOB-MRI.

CEUS	EOB-MRI				Total
	3	4	5	M	
3	17	13	0	0	30
4	6	13	5	1	25
5	0	27	22	2	51
M	0	12	8	1	21
Total	23	65	35	4	127

**Table 3:** Comparison of pathological results and category between CEUS and EOB-MRI.

	CEUS					EOB-MRI				
	3	4	5	M	Total	3	4	5	M	Total
HCC	2	12	50	18	82	2	44	34	2	82
ICC	0	0	0	2	2	0	1	0	1	2
NET	0	0	0	1	1	0	1	0	0	1
RN	2	1	0	0	3	0	2	0	1	3
DN	5	3	1	0	9	2	6	1	0	9
FNH	0	1	0	0	1	0	1	0	0	1
Hemangioma	0	1	0	0	1	0	1	0	0	2
Other benign	21	7	0	0	28	19	9	0	0	28
Total	30	25	51	21	127	23	65	35	4	127

HCC, hepatocellular carcinoma; ICC, intrahepatic cholangiocarcinoma; NET, neuroendocrine neoplasm; RN, regenerative nodule; DN, dysplastic nodule; FNH, focal nodular hyperplasia.

### 3.4 Diagnostic performance of CEUS and EOB-MRI LI-RADS for HCC ≤ 2 cm

The PPVs of CEUS and EOB-MRI LR-3, LR-4, and LR-5 for HCC were 6.7% (2/30), 48.0% (12/25), 98.0% (50/51), and 8.7% (2/23), 67.7%(44/65), and 97.1% (34/35), respectively. The diagnostic performance of CEUS and EOB-MRI LI-RADS LR-5 for HCC is shown in **Table 4**.

**Table 4:** The diagnostic performance of CEUS and EOB-MRI LR-5 for HCC.

	HCC		
	CEUS LR-5	EOB-MRI LR-5	p-value
TP	50	34	
TN	44	44	
FP	1	1	
FN	32	48	
Sensitivity (%)	61.0 (49.6, 71.6)	41.5 (30.7, 52.9)	<0.01
Specificity (%)	97.8 (88.2, 99.9)	97.8 (88.2,99.9)	0.31
PPV(%)	98.0 (87.7, 99.7)	97.1 (82.8, 99.6)	0.45
NPV(%)	57.9 (51.1, 64.4)	47.8 (43.2, 52.5)	0.10
AUC	0.79 (0.71-0.86)	0.70 (0.61-0.78)	<0.01

TP, true positive; TN, True negative; FP, False positive; FN, False negative; PPV, positive predict value; NPV, negative predict value.

There was no difference in PPVs, NPVs, and specificity between CEUS and EOB-MRI LR-5 for HCC. A total of 18 HCCs were classified to CEUS LR-M, while no ICC or neuroendocrine tumor was misclassified to CEUS LR-4 or LR-5. One ICC and one neuroendocrine tumor were misclassified to EOB-MRI LR-4.

**Figure 3:**
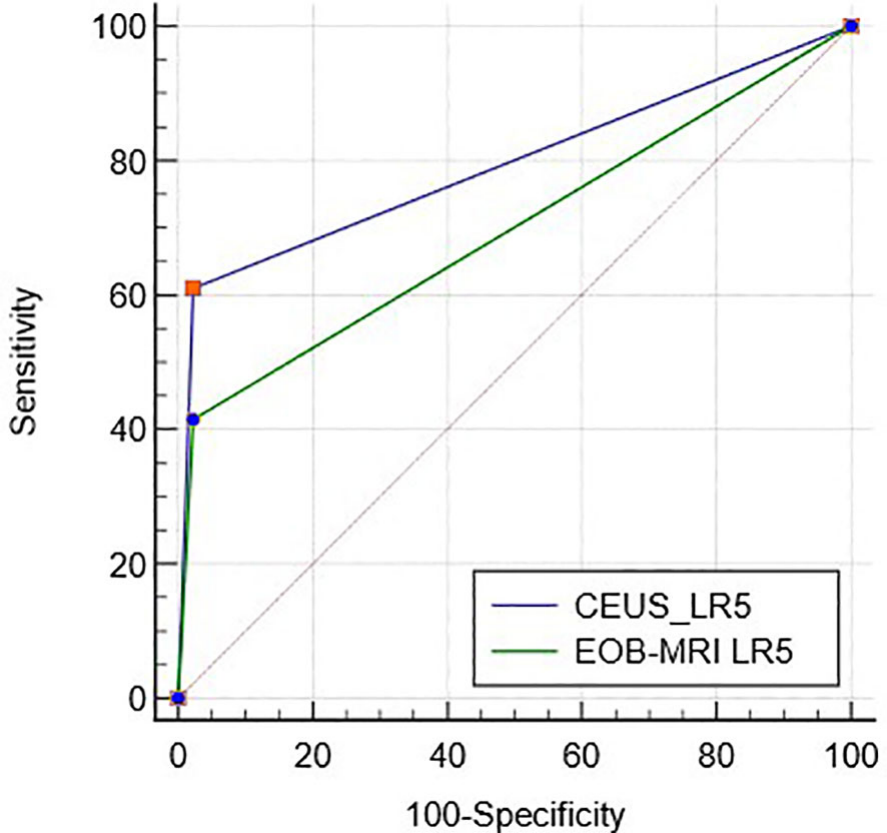
The ROC curve of CEUS and EOB-MRI LR-5.

ROC curves of CEUS and EOB-MRI LR-5 for HCC diagnosis are shown in **Figure 3**, and the AUC was 0.79 (95%CI: 0.71–0.86) and 0.70 (95%CI: 0.61–0.78) (p < 0.01).

### 3.5 Combination of CEUS and EOB-MRI LR-5 for HCC diagnosis

CEUS LR-5 and EOB-MRI LR-4/5 (criteria 1), CEUS LR-4/5 and EOB-MRI LR-5 (criteria 2), and criteria 1 or 2 (criteria 3) were used as diagnostic criteria for HCC, respectively. The diagnostic performance of criteria 1–3 for HCC is shown in **Table 5**. The diagnostic performance of criteria 3 shows no statistical difference with that of CEUS LR-5, while the sensitivity and AUC were higher than that of EOB-MRI LR-5 (p <0.01, **Supplementary Table S3**).

**Table 5:** The diagnostic performance of the combination of CEUS and EOB-MRI LI-RADS.

		HCC				
	*CEUS LR-5 + EOB-MRI LR4/5	**EOB-MRI LR5+ CEUS LR-4/5	***Criteria 1 or Criteria 2	p-value *vs.**	p-value *vs.***	p-value **vs.***
TP	48	26	52			
TN	44	44	43			
FP	1	1	2			
FN	34	56	30			
Sensitivity (%)	58.5 (47.1, 69.4)	31.1 (21.9, 42.6)	63.4 (52.0, 73.8)	<0.01	0.52	<0.01
Specificity (%)	97.8 (88.2, 99.9)	97.8 (88.2, 99.9)	95.6 (84.9, 99.5)	1	0.56	0.56
PPV(%)	97.6 (87.3-99.7)	96.3 (78.5,99.5)	96.3 (86.9, 99.0)	0.67	0.62	1
NPV(%)	56.4 (49.9, 62.7)	44.0 (40.2,47.8)	58.9 (51.7, 65.7)	0.10	0.76	0.05
AUC	0.78 (0.70-0.85)	0.65 (0.56,0.73)	0.80 (0.71,0.86)	<0.01	0.04	<0.01

TP, true positive; TN, true negative; FP, false positive; FN, false negative; PPV, positive predict value; NPV, negative predict value.

*means CEUS LR-5 + EOB-MRI LR4/5, ** means EOB-MRI LR5+ CEUS LR-4/5, *** means CEUS LR-5 + EOB-MRI LR4/5 or EOB-MRI LR5+ CEUS LR-4/5 (Criteria 1 or Criteria 2).

**Figure 4:**
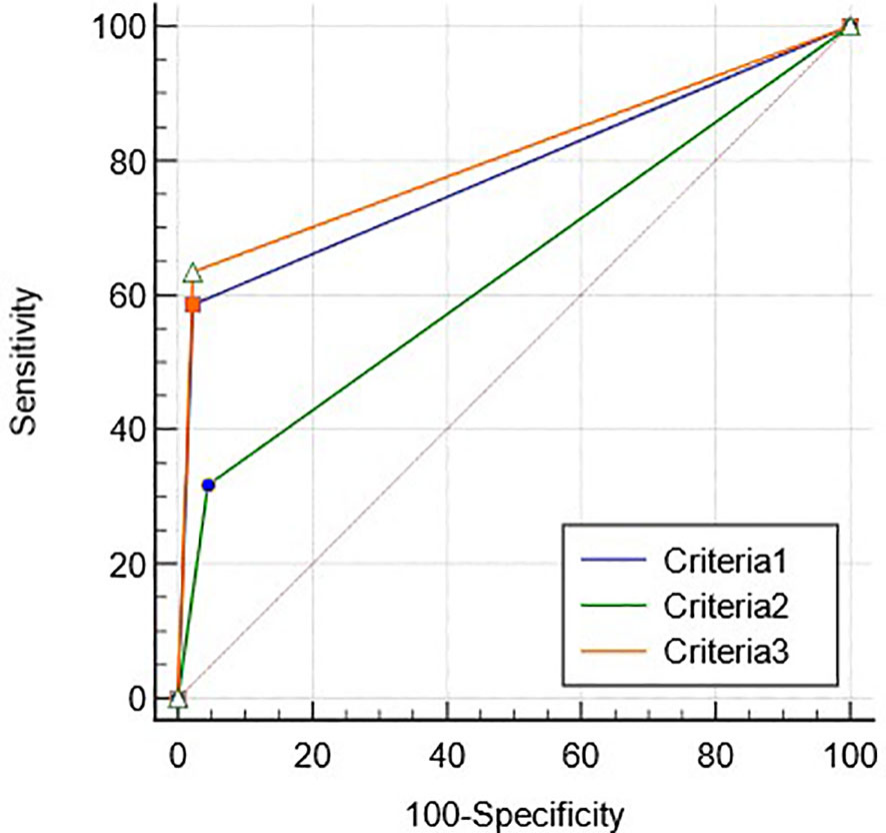
The ROC curve of the combination criteria 1–3 for diagnosing HCC.

The ROC curves of criteria 1–3 for diagnosing HCC are shown in **Figure 4**, and the AUCs were 0.78 (95%CI: 0.70–0.85), 0.65 (95%CI: 0.76–0.73) and 0.80 (95%CI: 0.71–0.86), respectively (p < 0.05).

## 4 Discussion

Early detection of HCC when it is amenable to curative therapy could reduce all-cause mortality. It is known that lesions with the typical enhancement pattern of HCC on imaging can be diagnosed noninvasively by imaging. Therefore, the progress of imaging is crucial for the diagnosis and treatment of HCC. In order to make the results of this study have specific value for early diagnosis of HCC, liver nodules ≤2cm were included as our study object. The result showed that there was fair inter-modality agreement between EOB-MRI and CEUS LI-RADS category of liver nodules in patients at high risk for HCC (kappa=0.21), which was similar to the results of a previous retrospective study that compared CECT/EOB-MRI and CEUS (kappa=0.319) (9). Another retrospective study using extracellular contrast agents by German scholars also had a similar result (kappa=0.218) (10). The reason for the fair consistency in LI-RADS between the two imaging modalities lies in the different imaging techniques and contrast agents, especially for hepatobiliary specific MRI (11, 12). Gadoxetate disodium began to be absorbed by liver cells from 60 s to 90 s after the injection. Due to the dual metabolic pathway of this contrast agent, the hypointensity lesions on the transitional phase is different from that of traditional CECT/MRI, so the washout of EOB-MRI was only limited to hypointensity on portal venous phase. Hypointensity on the hepatobiliary phase is taken as an auxiliary feature (13). There was also good inter-observer agreement for the LI-RADS of the two different imaging modalities. For EOB-MRI, there was a high inter-observer in our study (kappa=0.76), while it was reported in the previous studies that the inter-observer agreement was moderate (kappa= 0.405–0.518) (14). However, a recently published systematic review and meta-analysis of CEMRI inter-observer agreement showed that the inter-observer agreement of LI-RADS was similar to our study (kappa =0.7) (15).

Although LI-RADS is not just a diagnostic tool, its diagnostic performance has always been concerned, especially that of LR-5 for HCC. In this study, CEUS LR-5 showed high PPV and specificity for HCC ≤ 2 cm, which was consistent with the purpose of LI-RADS and similar to results of previous study (16–19). In our study, EOB-MRI LR-5 also showed high PPV and specificity for HCC ≤ 2 cm, similar to previous studies (20, 21). The PPV and specificity of EOB-MRI LR-5 in the diagnosis for HCC were not statistically significant compared with CEUS, but there were differences between the two AUCs, in which CEUS LR-5 was better than EOB-MRI LR-5. A retrospective study comparing the diagnostic performance of CEUS with CECT/MRI LR-5 showed that CEUS was superior to EOB-MRI (AUC: 0.994 vs. 0.760); the lower sensitivity of EOB-MRI LR-5 is one of the reasons why EOB-MRI LR-5 AUCs are inferior to CEUS. The sensitivity of EOB-MRI LR-5 for HCC have been reported differently (45.0%–67.3%) (20–22), which may be related to selection bias and different reference standard in each study. An important reason for the lower sensitivity of EOB-MRI LR-5 in this study is that LR-4 contains a high proportion of HCC (67.7%), accounting for 53.6% of all HCCs. The fundamental reason is also related to the LI-RADS criteria of EOB-MRI, that is, to be classified as LR-5, there must be washout in the portal venous phase or enhanced capsule. However, the washout onset of early HCC is usually late. A prior study showed that adding transitional phase hypointensity as washout helped diagnose HCC as LR-5; 13.7% (13 out of 95) HCC would be reclassified to LR-5 category, increasing the sensitivity of LR-5 category without changing the specificity (23).

Therefore, we found that both CEUS and EOB-MRI LR-4 had a high proportion of HCC (48.0% and 66.7%). We suggest that new diagnostic criteria for HCC by combining CEUS with EOB-MRI LI-RADS in order to improve sensitivity and specificity in diagnosing HCC. The results of this study showed that when we use CEUS LR-5 and EOB-MRI LR-4/5 or CEUS LR-4/5 and EOB-MRI LR-5 as diagnostic criteria for HCC, a sensitivity of 63.4% and a specificity of 95.6% could be obtained for HCC smaller than 2 cm. Using this criteria, four cases of CEUS LR-4 and EOB-MRI LR-5 lesions were correctly diagnosed as HCC. Our previous study used CEUS to reclassify CECT/MRI LR-3/4 nodules, which also improved the diagnostic performance of HCC (24). This diagnostic criterion was easy to use in clinical work, and this criterion is also consistent with the addition of another imaging examination when there is one imaging uncertainty of HCC diagnosis in the guidelines (25, 26). In our study, there were three non-HCC malignancies (two ICC and one neuroendocrine tumor), all of which were classified as CEUS LR-M. One ICC was classified as EOB-MRI LR-4 and the other two as EOB-MRI LR-M. Due to the small sample size of non-HCC malignancies, the diagnostic performance of LR-M was not analyzed.

There are some limitations in this study: (1) LR-1 and LR-2 nodules were not included because they were considered benign after receiving EOB-MRI or CEUS, but no other imaging examination or CECT/MRI was performed; (2) all the included nodules were visible nodules on ultrasound. “Pseudo-lesions”, such as the artery-portal venous shunt (APS), visible by EOB-MRI were not included. (3) The sample size was small, and the proportion of nodules in each category may have bias.

In conclusion, CEUS and EOB-MRI showed fair inter-modality agreement in the category of LI-RADS in nodules ≤2cm. The inter-observer agreement of CEUS and EOB-MRI LI-RADS were substantial. CEUS and EOB-MRI LR-5 have equally good positive predictive value and specificity for HCC ≤ 2 cm, and combining these two modalities can better diagnose HCC ≤ 2 cm.

## Data availability statement

The original contributions presented in the study are included in the article/**Supplementary Material**. Further inquiries can be directed to the corresponding authors.

## Ethics statement

The studies involving humans were approved by Ethics Committee of Tianjin Third Central Hospital (IRB2019-019-01). The studies were conducted in accordance with the local legislation and institutional requirements. The participants provided their written informed consent to participate in this study.

## Author contributions

ZQ: Data curation, Formal analysis, Methodology, Writing – original draft, Writing – review & editing. YZ: Conceptualization, Investigation, Methodology, Writing – review & editing. XZ: Writing – review & editing. JD: Conceptualization, Investigation, Writing – review & editing. HZ: Formal analysis, Investigation, Writing – review & editing. YW: Investigation, Writing – review & editing. LZ: Investigation, Methodology, Writing – review & editing. CC: Investigation, Writing – review & editing. XJ: Conceptualization, Investigation, Supervision, Writing – review & editing.
