# Supplementary material for: The comparison of contrast-enhanced ultrasound and gadoxetate disodium-enhanced MRI LI-RADS for nodules ≤2 cm in patients at high risk for HCC: a prospective study
Source: Front Oncol. 2024 May 7;14:1345981. doi: 10.3389/fonc.2024.1345981 (PMC11106436; doi:10.3389/fonc.2024.1345981)
Supplement: Supplementary file 1 [file Table_1.docx]

Supplementary Table 1 Inter-observer agreement of CEUS LI-RADS

| R1  R2 | 3 | 4 | 5 | M | Total |
| --- | --- | --- | --- | --- | --- |
| 3 | 29 | 0 | 0 | 0 | 29 |
| 4 | 1 | 19 | 5 | 0 | 25 |
| 5 | 0 | 5 | 43 | 4 | 52 |
| M | 0 | 0 | 4 | 17 | 21 |
| Total | 30 | 24 | 52 | 21 | 127 |

R1 = Reader1；R2 = Reader2

Supplementary Table 2 Inter-observer agreement of EOB-MRI LI-RADS

| R1  R2 | 3 | 4 | 5 | M | Total |
| --- | --- | --- | --- | --- | --- |
| 3 | 20 | 3 | 0 | 0 | 23 |
| 4 | 1 | 57 | 6 | 0 | 64 |
| 5 | 0 | 7 | 28 | 0 | 35 |
| M | 0 | 2 | 0 | 3 | 5 |
| Total | 21 | 69 | 34 | 3 | 127 |

R1 = Reader1；R2 = Reader2

Supplementary Table 3 The diagnostic performance of the combination of CEUS and EOB-MRI LI-RADS

|  |  | HCC | | | |  |
| --- | --- | --- | --- | --- | --- | --- |
|  | *CEUS LR-5 | | **EOB-MRI LR5 | ***Criteria 3 | *P* Value  *vs*** | *P* Value  **vs*** |
| TP | 50 | | 34 | 52 |  |  |
| TN | 44 | | 44 | 43 |  |  |
| FP | 1 | | 1 | 2 |  |  |
| FN | 32 | | 48 | 30 |  |  |
| Sensitivity (%) | 61.0  (49.6, 71.6) | | 41.5  (30.7, 52.9) | 63.4  (52.0, 73.8) | 0.74 | ＜0.01 |
| Specificity (%) | 97.8  (88.2, 99.9) | | 97.8  (88.2,99.9) | 95.6  (84.9, 99.5) | 0.56 | 0.56 |
| PPV(%) | 98.0  (87.7, 99.7) | | 97.1  (82.8, 99.6) | 96.3  (86.9, 99.0) | 0.59 | 0.83 |
| NPV(%) | 57.9  (51.1, 64.4) | | 47.8  (43.2, 52.5) | 58.9  (51.7, 65.7) | 0.90 | 0.16 |
| AUC | 0.79  (0.71-0.86) | | 0.70  (0.61-0.78) | 0.80  (0.71,0.86) | 0.94 | ＜0.01 |
